# In Vitro Characterization of Inhalable Cationic Hybrid Nanoparticles as Potential Vaccine Carriers

**DOI:** 10.3390/ph14020164

**Published:** 2021-02-18

**Authors:** Iman M. Alfagih, Kan Kaneko, Nitesh K. Kunda, Fars Alanazi, Sarah R. Dennison, Hesham M. Tawfeek, Imran Y. Saleem

**Affiliations:** 1 School of Pharmacy and Biomolecular Sciences, Liverpool John Moores University, Liverpool L3 3AF, UK; fagih@ksu.edu.sa (I.M.A.); kanKaneko@gmail.com (K.K.); kundan@stjohns.edu (N.K.K.); 2 Department of Pharmaceutics, College of Pharmacy, King Saud University, Riyadh 11451, Saudi Arabia; 3 Department of Pharmaceutical Sciences, College of Pharmacy and Health Sciences, St. John’s University, Jamaica, NY 11439, USA; 4 Kayali Chair for Pharmaceutical Industries, Department of Pharmaceutics, College of Pharmacy, King Saud University, Riyadh 11451, Saudi Arabia; afars@ksu.edu.sa; 5 Faculty of Clinical and Biomedical Sciences, School of Pharmacy and Biomedical Sciences, University of Central Lancashire, Preston PR1 2HE, UK; srdennison1@uclan.ac.uk; 6 Department of Industrial Pharmacy, Faculty of Pharmacy, Assiut University, Assiut 71526, Egypt; heshamtawfeek79@gmail.com

**Keywords:** protein, hybrid nanoparticles, nanocomposite microparticles, chitosan, pulmonary delivery, inhalation, dry powder, spraydrying, vaccine

## Abstract

In this study, PGA-co-PDL nanoparticles (NPs) encapsulating model antigen, bovine serum albumin (BSA), were prepared via double emulsion solvent evaporation. In addition, chitosan hydrochloride (CHL) was incorporated into the external phase of the emulsion solvent method, which resulted in surface adsorption onto the NPs to form hybrid cationic CHL NPs. The BSA encapsulated CHL NPs were encompassed into nanocomposite microcarriers (NCMPs) composed of l-leucine to produce CHL NPs/NCMPs via spray drying. The CHL NPs/NCMPs were investigated for in vitro aerosolization, release study, cell viability and uptake, and stability of protein structure. Hybrid cationic CHL NPs (CHL: 10 mg/mL) of particle size (480.2 ± 32.2 nm), charge (+14.2 ± 0.72 mV), and BSA loading (7.28 ± 1.3 µg/mg) were produced. The adsorption pattern was determined to follow the Freundlich model. Aerosolization of CHL NPs/NCMPs indicated fine particle fraction (FPF: 46.79 ± 11.21%) and mass median aerodynamic diameter (MMAD: 1.49 ± 0.29 µm). The BSA α-helical structure was maintained, after release from the CHL NPs/NCMPs, as indicated by circular dichroism. Furthermore, dendritic cells (DCs) and A549 cells showed good viability (≥70% at 2.5 mg/mL after 4–24 h exposure, respectively). Confocal microscopy and flow cytometry data showed hybrid cationic CHL NPs were successfully taken up by DCs within 1 h of incubation. The upregulation of CD40, CD86, and MHC-II cell surface markers indicated that the DCs were successfully activated by the hybrid cationic CHL NPs. These results suggest that the CHL NPs/NCMPs technology platform could potentially be used for the delivery of proteins to the lungs for immunostimulatory applications such as vaccines.

## 1. Introduction

Vaccination via the lungs is an attractive proposition, as it mimics the natural infection route of pulmonary pathogens and can lead to local and systemic immune protection [1,2]. Administration via inhalation offers the additional advantage of eliminating syringes and needles, and hence, their storage and disposal, resulting in the exclusion of blood-borne infections. The lung also offers many advantages from a delivery perspective, such as antigen presenting cells, including DCs, within the lungs, the large surface area for drug absorption, thin epithelium in the alveolar lung tissue, lower enzymatic activity than the gastrointestinal system, and large vascularization that can enable the efficient systemic delivery of drugs [2]. In order to ensure sufficient induction of immune responses, the vaccine formulation must demonstrate appropriate pulmonary deliverability, biocompatibility, and immunogenicity Biodegradable nanoparticles (NPs) have received considerable attention as potential vaccine delivery vehicles. Poly(glycerol adipate-co-ω-pentadecalactone) (PGA-co-PDL) is one such copolymer which can form NPs to provide a biocompatible delivery vehicle for encapsulated antigen. The hydrophobic nature of the polymer, compared to PLGA, is postulated to improve uptake by antigen presenting cells to improve resulting immune responses. In addition to hydrophobicity, the size and surface charge of NPs have a significant effect on cellular uptake, with cationic particles postulated to interact with cell membranes which are negatively charged more effectively through ionic interactions [3]. The positive charge is subsequently also generally associated with greater immunogenicity and vaccine response [3]. Many research groups, including ours, have documented techniques of modifying the surface charge of NPs using didodecyldimethylammoniumbromide (DMAB) to form hybrid NPs [4,5,6]. However, the use of these cationic surfactants and lipids has been shown to generate toxicity [7,8,9]. Here, we focused on the use of the polymer chitosan.

Chitosan is a cationic polysaccharide which is biodegradable and biocompatible, with low toxicity, and has been used in various ways, such as as a solution, gel, powder, microparticles, and nanoparticles. The cationic charge from the amine groups is postulated to be responsible for mucoadhesion, opening of tight junctions, cell uptake, and cGAS activation, which lead to immunogenic effects. These adjuvant effects of chitosan are useful for antigens which do not possess intrinsic immunogenicity. However, it possesses low water solubility. This can be overcome by chemically modifying the polymer backbone to produce derivatives with enhanced water solubility, interaction with other substances, as well as other properties such as antimicrobial activity [10]. Water-soluble chitosan hydrochloride (CHL) is one example [11] and, as with other positively charged polymers, has the potential to contribute to toxicity at high concentrations. The toxicity of cationic materials has been postulated to result from inflammatory responses caused by the binding to and subsequent impairment of Na+/K+-ATPase [12]. There is also the possibility that the resulting cell necrosis is the exact same mechanism of how immunostimulation is derived from cationic materials. Although the toxicity has been a concern regarding tolerance, this can be overcome by decreasing the amount of CHL in the formulation by adsorbing onto anionic NPs, due to the interfacial adsorption phenomenon [13].

In order to formulate these positively charged, hybrid cationic CHL NPs into an inhalable form, the particles can be integrated into microcarriers forming nanocomposite microparticles (NCMPs) with an aerodynamic size of 1 to 5 µm [14]. These systems are designed to be within the size range which is favorable for deposition into the lungs when inhaled as a dry powder, and undergo dissolution in the fluid lining the lung, releasing the NPs from the inert carrier [15]. The released NPs would then be candidates for uptake and interaction with the dendritic cells (DCs), which are essential antigen presenting cells in the lungs, and this leads to the induction of an immune response which results in adaptive immune protection.

In this study, we investigated the influence of CHL concentration and determine the adsorption mechanism onto the surface of PGA-co-PDL NPs with encapsulated model protein BSA to form hybrid cationic CHL NPs. Furthermore, the BSA encapsulated CHL NPs were encompassed into NCMPs composed of L-leucine to produce CHL NPs/NCMPs via spray drying. The CHL NPs/NCMPs possessed suitable characteristics for dry powder pulmonary administration. The structure of BSA was determined using circular dichroism, and SDS-PAGE was used to determine if the BSA was fragmented during the CHL NPs/NCMPs fabrication process. Maintaining the structure of proteins during CHL NPs/NCMPs fabrication and release is important to maximize and preserve their activity. Lastly, the interaction of the CHL NPs with DCs and the subsequent changes in surface marker expression were investigated in an in vitro assay to determine the practicability of using these particles for vaccine applications.

## 2. Results

### 2.1. Chitosan Hydrochloride Adsorption Isotherm Model

The nature of chitosan adsorption to the NP surface and calculation of the maximum adsorption capacity is possible through several known adsorption models, including Langmuir, BET, and Freundlich. Langmuir describes the adsorption process onto the surface of the adsorbent as a continuous function of the initial concentration of the drug. The BET model was considered as an extension of Langmuir in which the theory for monolayer molecular adsorption is extended to multilayer adsorption. Moreover, Freundlich discovered the adsorption isotherm stating the relationship between the concentration of a solute on the surface of an adsorbent, to that in the liquid with which it is in contact.

**Figure 1:**
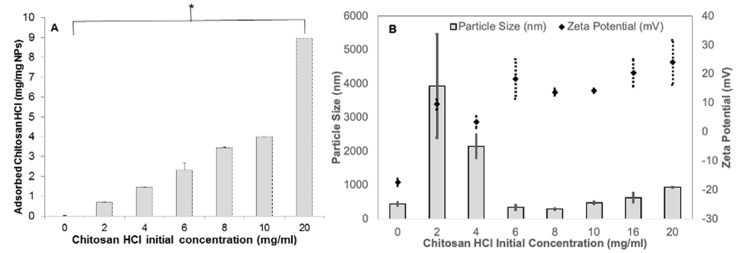
The relationship of CHL concentration to the amount absorbed on PGA-co-PDL NPs (**A**). Zeta potential and particle size of hybrid cationic CHL NPs (**B**) (*n* = 3, *p* < 0.05 is indicated by *).

Adsorption of CHL onto the surface of NPs significantly increased with the CHL concentration (0–20 mg/mL) (Figure 1A). The mass of CHL adsorbed per mg of NPs increased from 0.69 ± 0.01 to 8.97 ± 0.03 mg with increase in CHL concentrations from 2 to 20 mg/mL, respectively. The CHL concentration of 20 mg/mL produced the largest mass of adsorbed CHL per mg NPs (8.97 mg) (*p* < 0.05, ANOVA/Tukey’s comparison; all values were significant to one another).

**Figure 2:**
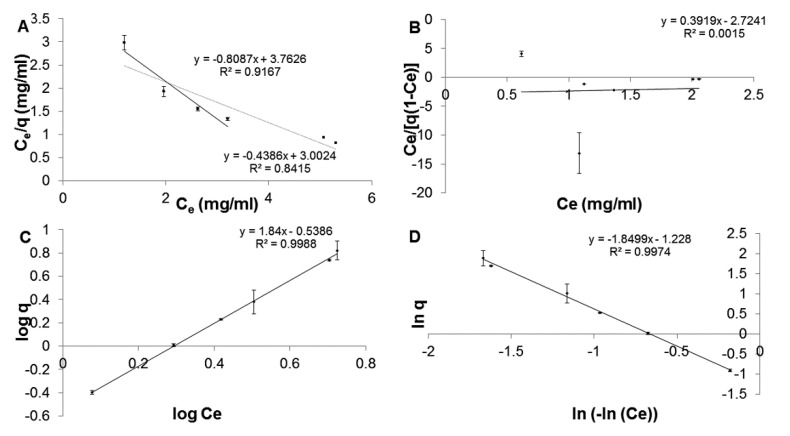
Linear description of CHL adsorption onto PGA-co-PDL NPs. Ce is the residual CHL concentrations in the suspension at equilibrium (mg/mL) and q is the amount of adsorbed CHL per unit weight of NPs. All models (**A**) Langmuir, (**B**) BET, (**C**) Freundlich (**D**) Halsey were fit utilizing concentrations of 2–20 mg/mL; however, the Langmuir model was also fit to low concentrations (0–8 mg/mL) (black) (*n* = 3).

The CHL adsorption values were fed into the isotherm equations and we observed that the CHL adsorption did not comply with the Langmuir model for the concentrations investigated (Figure 2A); however, at low concentrations (0–8 mg/mL), compliance with the Langmuir model was observed. For example, CHL concentration (8 mg/mL) resulted in a monolayer adsorption capacity (q_m_) of 1.2366 mg/mg NPs. In addition, CHL adsorption with the BET model also had a poor fit (Figure 2B). The Freundlich (Figure 2C) and Halsey (Figure 2D) multilayer adsorption models had a good correlation (r^2^ > 0.99), suggesting suitable fits for CHL adsorption onto NPs, and the corresponding adsorption parameters were calculated with Freundlich isotherm model being the most suitable (Table 1).

### 2.2. Characterization of Nanoparticles

The NPs with no CHL produced a negative (−17.44 ± 1.2 mV) zeta potential, whereas all NPs prepared with different CHL concentrations were positive (Figure 1B) (*p* < 0.05, ANOVA/Tukey’s comparison). Moreover, there was an increase in zeta-potential, which correlated with a rise in CHL concentrations (2–20 mg/mL) up to +24.0 ± 7.7 mV (Figure 1B).

The particle size (Figure 1B) of non-chitosan coated NPs was 445 ± 46.8 nm, which increased (3934 ± 1533.7 and 2148 ± 352.6 nm) significantly (*p* < 0.05, ANOVA/Tukey’s comparison) upon addition of CHL (2 to 4 mg/mL), while at higher concentrations of CHL (more than 6, 10, 16 mg/mL), the particle size did not significantly change (*p* > 0.05, ANOVA/Tukey’s comparison). Furthermore, there was no significant difference in the zeta-potential of NPs after 6 mg/mL (*p* > 0.05, ANOVA/Tukey’s comparison). Moreover, 10 mg/mL achieved high CHL adsorption compared to other concentrations without significant change to size (480.23 ± 32.2 nm) compared to non-chitosan coated NPs. Consequently, CHL at 10 mg/mL was selected for further investigations. Both the protein loading (43.67 ± 2.3 µg/mg) and EE% (48 ± 2.6%) of CHL NPs were significantly higher (*p* < 0.05, ANOVA/Tukey’s comparison) in comparison with the non-chitosan coated NPs, which exhibited 7.28 ± 1.3 µg/mg and 4.9 ± 2.1%, respectively.

### 2.3. Characterization of Nanocomposite Microparticles

**Figure 3:**
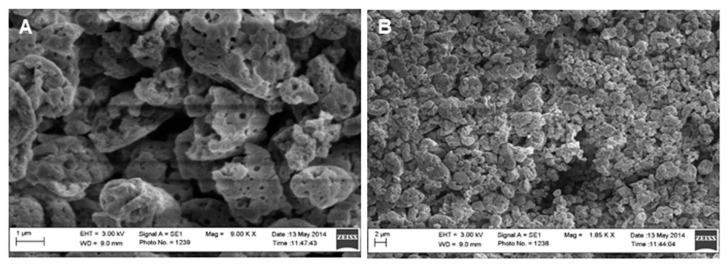
Scanning electron microscopy images of cationic CHL NPs/NCMPs: (**A**) 1 µm scale and (**B**) 2 µm scale.

Cationic CHL NPs/NCMPs were produced by spray drying (yield: 50.9 ± 2.3%) where the selected hybrid cationic CHL NPs were dispersed in L-leucine, which was used as a carrier and dispersing agent to improve powder flow upon inhalation. Images of the cationic CHL NPs/NCMPs indicated an irregular and porous morphology (Figure 3A,B). The size of hybrid cationic CHL NPs following dispersion of spray-dried cationic CHL NPs/NCMPs in distilled water was 490 ± 17 nm.

In terms of aerodynamic properties, the geometric particle size of cationic CHL NPs/NCMPs formulation was 5.52 ± 0.64 µm, the tap density was 0.08 ± 0.002 gcm^−3^. The theoretical aerodynamic diameter (d_ae,_ 1.56 ± 0.19 µm) was calculated from geometric particle size and tap density. Protein deposition data obtained from NGI resulted in MMAD of 1.49 ± 0.29 µm, FPD 32.51 ± 6.67 µg and FPF 46.79 ± 11.21%.

### 2.4. In Vitro Release Analysis

**Figure 4:**
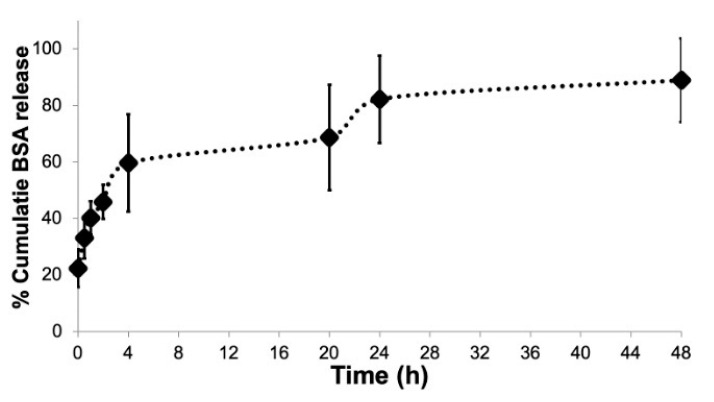
In vitro release of BSA from cationic CHL NPs/NCMPs in PBS buffer at 37 °C (*n* = 3).

In vitro release studies were carried out on cationic CHL NPs/NCMPs formulations with data expressed as cumulative percentage protein released over time (Figure 4). The formulation presented a biphasic release profile, with the % cumulative protein released from cationic NPs/NCMPs at time zero (22.42 ± 6.7%) interpreted as surface-associated protein. The % cumulative protein released at the end of 4 h (59.70 ± 17.2%) was interpreted as the initial burst, which then transgressed into a continuous sustained phase to 48 h (Figure 4). The cationic NPs/NCMPs formulations showed a relatively high cumulative release, with nearly 88.93 ± 14.8% of protein released after 48 h (Figure 4). In vitro release kinetics model (zero, first order, and the Higuchi rate) were used to identify the mechanism of protein release (Table 2). BSA was released in compliance with a combination of first order (r^2^ value of 0.919, release rate constant k_1_ (h^−½^) of −0.016) and Higuchi diffusion (r^2^ value of 0.928, release rate constant k_1_ (h^−½^) of 9.316) (Table 2).

### 2.5. Analysis of BSA Structure

**Figure 5:**
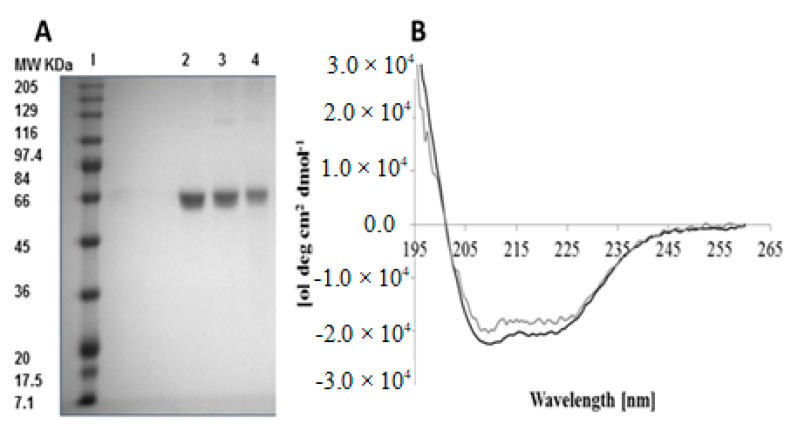
SDS-PAGE to evaluate the BSA stability released from cationic CHL NPs/NCMPs (**A**). Lanes represent, molecular weight (MW) standard markers, BSA (MW 66,000) (**1**), BSA standard (**2**), BSA released from hybrid cationic CHL NPs (**3**), and BSA released from cationic CHL NPs/NCMPs (**4**). Difference in band intensity was due to different loading. (The CD spectra of BSA released from cationic CHL NPs/NCMPs (grey) and BSA standard (black) (**B**) (*n* = 3).

The primary structure of BSA was examined by SDS-PAGE analysis after release from cationic NPs and cationic NCMPs. The molecular weight marker and the protein standard exposed a band at ~66 KDa (Figure 5A). The BSA released from hybrid cationic CHL NPs (Lane 3) and cationic CHL NPs/NCMPs (Lane 4) indicated comparable band patterns to the standard BSA. The bands provided evidence that the BSA in both formulation samples did not degrade nor formed aggregates during the formulation process. In other words, the BSA encapsulated in NPs preserved its primary structure.

The secondary structure of released protein from cationic CHL NPs/NCMPs was investigated using CD. The CD spectra (Figure 5B) presents two minima between 221–222 and 209–210 nm. Furthermore, at 195 nm, a maximum was observed for standard BSA and BSA released, characteristic of an α-helical structure. The analysis further revealed that the BSA standard was primarily α-helical (51.5% helicity, Table 3), which was reduced (circa 43% helical, Table 3) for released BSA from cationic CHL NPs/NCMPs. Additionally, comparing released BSA with standard BSA (Table 3) indicated an 8.5% reduction in α-helical content, an increase of 7.5% in β-sheet content, a decrease of 2% in the turns content, and an increase of 3% in the random coils’ content.

### 2.6. Cell Viability, Nanoparticle Uptake, and Surface Marker Upregulation by DCs

**Figure 6:**
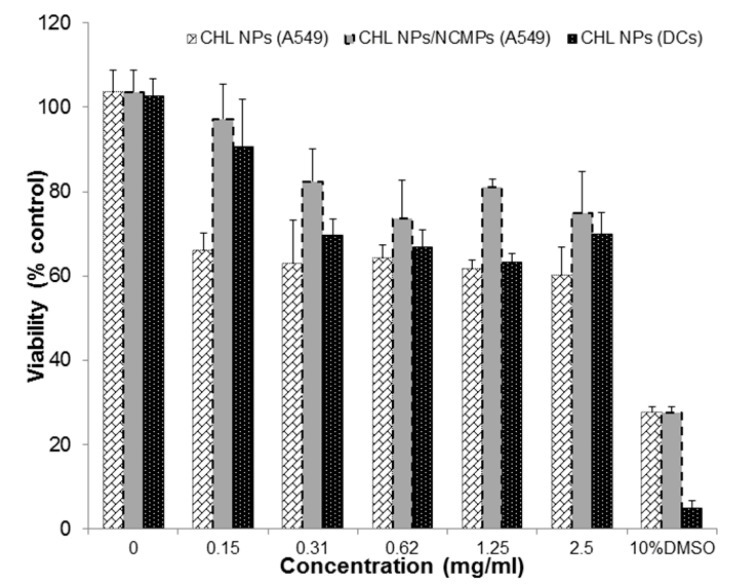
MTT assay to determine cell viability of A549 cells after 24 h of exposure to hybrid cationic CHL NPs and cationic CHL NPs/NCMPs, and DCs after 4 h of exposure to hybrid cationic CHL NPs (*n* = 3).

**Figure 7:**
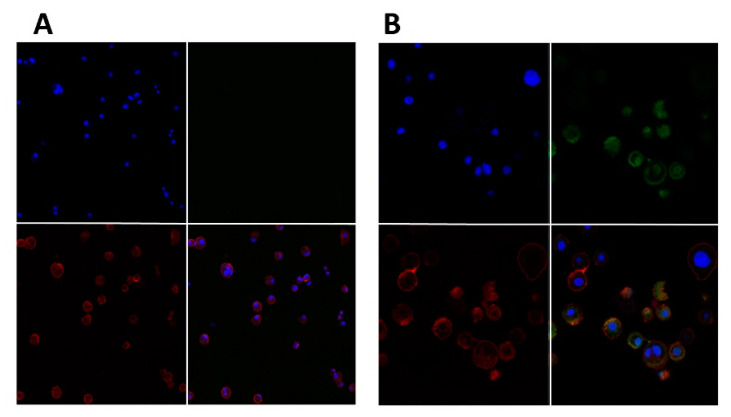
Confocal microscopy images of hybrid cationic CHL NPs uptake by DCs after 1 h incubation. DCs incubated without hybrid cationic CHL NPs at 20× (**A**), DCs incubated with hybrid cationic CHL NPs at 63× (**B**). Red channel for WGA TR: cell membrane, blue channel for DAPI: nucleus, and green channel for FITC-BSA: hybrid cationic CHL NPs incorporating FITC-BSA.

**Figure 8:**
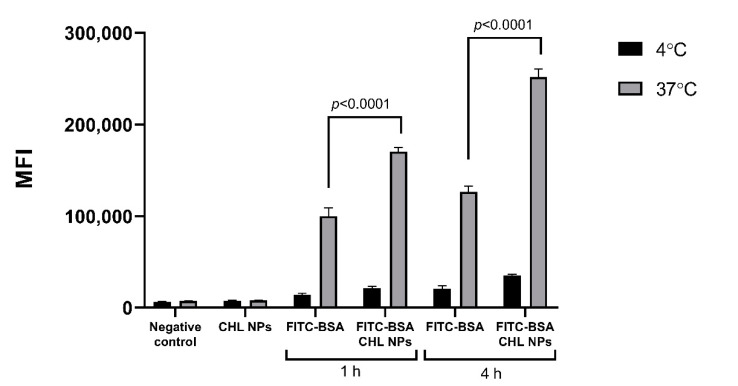
The association/uptake of FITC-BSA in cationic CHL NPs, by DCs. DCs were incubated with FITC-BSA incorporated CHL NPs for either 1 or 4 h, at either 4 or 37 °C (*n* = 4).

The hybrid cationic CHL NPs and cationic CHL NPs/NCMPs presented a cell viability profile in A549 cells of 63.91 ± 0.87% and 78.85 ± 9.96%, respectively, at 2.5 mg/mL following 24 h of exposure (Figure 6). In addition, the hybrid cationic CHL NPs was examined following 4 h of exposure in DCs, showing a decrease in cell viability (69.82 ± 5.28%) with increasing concentration up to 2.5 mg/mL. CLSM confirmed the uptake of FITC-BSA loaded hybrid cationic CHL NPs by DCs after 1 h incubation (Figure 7A,B). The visual cationic CHL NP uptake result was further supported by the flow cytometry data showing an increase in the FITC signal of the cells 1 and 4 h after co-incubation (Figure 8, *p* < 0.01). The cells incubated with the FITC-BSA loaded hybrid cationic CHL NPs also had greater median fluorescence intensity (MFI) compared to the FITC-BSA alone, suggesting that the particulate form is able to deliver greater quantity of the protein to the DCs. The FITC signal from the DCs incubated at 4 °C was much lower compared to 37 °C, suggesting that the uptake and adhesion process is temperature-dependent. As energy-dependent uptake and passive diffusion mechanisms are inhibited at 4 °C, this observation could suggest that the measured signal from the cells at 37 °C originates from actual uptake into the cells, as opposed to just adhesion on the cell surface [16].

**Figure 9:**
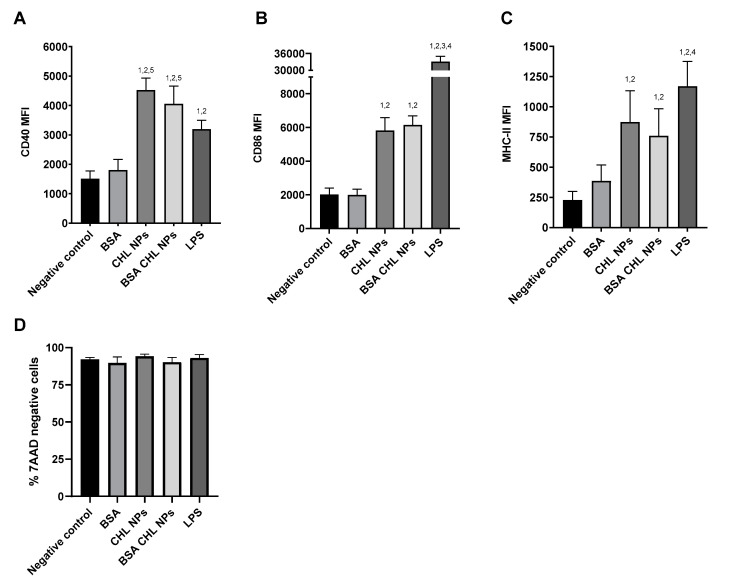
Upregulation of cell surface CD40 (**A**), CD86 (**B**), and MHC-II (**C**) on DCs, and cell viability according to 7AAD staining (**D**), after incubation with BSA incorporated hybrid cationic CHL NPs for 24 h (*n* = 6). The indicated groups exhibited significantly greater MFI (*p* < 0.05) compared to negative control. (**1**), BSA (**2**), CHL NPs (**3**), BSA CHL NPs (**4**), LPS (**5**).

DCs were shown to be activated by the hybrid cationic CHL NPs after 24 h, as shown by the increase in CD40, CD86, and MHC-II cell surface markers, compared to groups which were incubated with only the BSA or blank media (Figure 9). The incorporation of the BSA had little effect on the immunogenicity of the NPs, as shown by the lack of a significant difference between NP with/without BSA. During the incubation, the cells exhibited no significant detriment in viability according to 7AAD staining (Figure 9D).

## 3. Discussion

### 3.1. Chitosan Hydrochloride Adsorption Isotherm Model

Adsorption of CHL onto the PGA-co-PDL NPs was explored to change the surface charge to positive, and promote potential interactions of hybrid cationic CHL NPs with negatively charged cell membranes [17]. The amount of CHL adsorbed per mg of NPs increased with feed CHL concentrations (0–20 mg/mL), without reaching a steady state of mass adsorption (Figure 1).

The Langmuir model (Figure 2A): This model illustrated monolayer adsorption of adsorbate (CHL) on the adsorbent (PGA-co-PDL) where all adsorption sites are equivalent; there was no interaction between adsorbate molecules, and the adsorption was not governed by surface occupation [18]. The Langmuir monolayer model did achieve a good fit at low concentrations of CHL (0–8 mg/mL), but not at higher concentrations (10–20 mg/mL).

The BET model (Figure 2B): This was an expansion of the Langmuir model (monolayer model) to incorporate multilayer adsorption. The layers following the first layer consist of condensed adsorbate molecules, with the assumption of equal energies of adsorption, indicating uniform surface characteristics. Furthermore, BET only considers short distance interactions between the CHL and PGA-co-PDL molecules. The data obtained did not fit this model.

The Freundlich model (Figure 2C): This model considers multilayer adsorption on a uniform and non-uniform surface [19]. The values obtained from the slope 1/n (0.33–1.18), was a measure for surface heterogeneity or the adsorption intensity. When the value becomes closer to zero it indicates a more heterogeneous surface. In this study, the calculated value of 1/n was greater than 1, which indicated evidence of a cooperative adsorption [20]; hence, the nonlinear adsorption was mostly represented by Freundlich model.

The Halsey isotherm model (Figure 2D): This explains multilayer adsorption of an adsorbate on a heterogeneous surface, which was demonstrated by the relationship involving the adsorbate mass (*q*) and the adsorbate free concentration at equilibrium (*C_e_*). Consequently, this model comprised three segments. The first and second segment consisted of cooperative adsorption on a non-uniform surface, whereas the third segment was associated with cooperative multilayer adsorption consisting of van der Waals forces at some length from the surface [21].

The most important factor was the electrostatic forces between the positive CHL and negative PGA-co-PDL charge in the development of the first adsorption layer. When the CHL concentration increased, the following layers of CHL were adsorbed without interaction with the NPs surface. Hence, the CHL molecules would repel each other due to similar charge, although CHL molecules could have interacted via van der Waal’s forces, hydrophobic interactions, and hydrogen bonds. Mostly, the NPs large surface area and energy had a significant involvement in CHL multilayer adsorption [22].

### 3.2. Nanoparticle Characterization

The particle size of hybrid cationic CHL NPs increased significantly at the lower concentrations of CHL (2 to 4 mg/mL), whereas when the CHL concentrations increased (6, 10, 16 mg/mL), the particle size did not significantly increase (Figure 1B). This could be due to the zeta potential of the particles being closest to neutral at the low chitosan concentrations, which promotes aggregation due to lower repulsive forces. There is also the possibility that complete coating of the NPs at above 4 mg/mL could promote steric hinderance and prevention of aggregation. CHL NPs displayed a positive charge at all tested CHL concentrations due to the electrostatic interactions between PGA-co-PDL (negative groups) and CHL (positive groups). Few amino groups from the chitosan would be needed to neutralize the PGA-co-PDL negative charge, while the residual free CHL amino groups would contribute to the zeta potential increase [23]. The continued adsorption of CHL onto NPs produced no change in the zeta-potential at high CHL concentrations (16–20 mg/mL) (Figure 1A), as the apparent surface charge per surface area remained consistent [24].

### 3.3. Nanocomposite Microparticles Characterization

Cationic CHL NPs/NCMPs exhibited a porous and irregular morphology (Figure 3A,B), which was further indicated by the tap density (0.08 ± 0.002 gcm^−3^). In addition, the size of hybrid cationic CHL NPs suitable for DC uptake was confirmed after dispersing cationic CHL NPs/NCMPs in distilled water [17]. The cationic CHL NPs/NCMPs had an MMAD (1.49 ± 0.29 µm) suitable for pulmonary delivery suggesting deposition within the bronchiole-alveolar region [25]. However, as the formulation is designed for the delivery of vaccine candidates, generally, the dose required for immune response is low and can be adjusted; hence, the FPD and FPF are suitable [15].

The release of BSA from cationic CHL NPs/NCMPs (Figure 4) occurred by first order and Higuchi diffusion model (Table 2). The first order kinetics portrayed the BSA release that was not encapsulated within the NPs but was on or close to the surface and, hence, available for dissolution. This occurred instantly when the NCMPs was placed into the PBS medium and is most likely associated with the CHL NPs not being incorporated within the microcarrier system [26]. The cationic CHL NPs/NCMPs formulations exhibited high cumulative protein release at the end of 48 h. The release pattern has been shown to be influenced by a combination of factors, including BSA loading and dispersion within NPs and presence of CHL on the surface of the NPs [27]. In addition, the CHL adsorbed on PGA-co-PDL NPs is water soluble, and thus, may not impede drug diffusion [26,28]. By assessing the release model of the CHL NPs/NCMPs, which was the Higuchi model, the remaining two phases of BSA release appeared to be a diffusion-limited process. The effective diffusivity of a drug is frequently increased exponentially due to the magnitude of polymer erosion or degradation. The second part is attributed to the CHL coat, which functions as a physical barrier that erodes, consequently slowing the BSA release. Erosion occurs due to the ability of CHL to absorb water, resulting in weakening the bonds between CHL and PGA-co-PDL followed by erosion of the NP coat, resulting in drug diffusion [29,30,31]. The third part is attributed to the degradation mediated through the hydrolysis of ester linkages in PGA-co-PDL backbone [32].

Standard BSA and the BSA released from hybrid cationic CHL NPs and cationic CHL NPs/NCMPs revealed similar bands in the SDS-PAGE gels (Figure 5). This confirmed that the primary structure of BSA entrapped in hybrid cationic CHL NPs and cationic CHL NPs/NCMPs samples was maintained during the formulation and manufacture. The secondary structure of BSA was assessed by CD (Figure 5B). Structural analysis was in good agreement with previous reports and presented that the BSA was predominantly α-helical [33]. In addition, the BSA released from NCMPs also verified the existence of α-helix and β-sheets, but they were modified in comparison with the BSA standard. This was related to the electrostatic interaction between the CHL (positive charge) and BSA (negative charge), and is most likely the cause for the slight increase in bands from CHL NPs and CHL NPs/NCMPs seen in the SDS-PAGE gel [34].

The changes in the secondary structure of BSA opens questions regarding the potential influence on the resulting immune response. Although the SDS-page indicates a lack of change to the main backbone of the protein, the changes in secondary structure could potentially lead to further changes to the tertiary structure. On the one hand, proteins are ultimately cleaved into antigenic peptides for loading onto the MHC molecules, which means that their post-cleavage structure is what will ultimately influence the immune response. On the other hand, the conformation stability is postulated to affect the intracellular fate of the antigen, including the unfolding and cleavage by proteases, which would affect how the antigens are loaded onto the MHC molecules [35]. In addition, the loss of tertiary structure fold stability can influence the conformational epitopes for B cell receptors. Recent studies have postulated the significance of B cell antigen presentation for the activation of naïve CD4+ T cell activation [36].

Although antigen conformation can influence the presentation pathway, there is also evidence from studies on popular adjuvants, such as alum, o/w emulsions, and polymers, that show that conformational changes do not always affect bioactivity [37]. The interaction between adjuvant and antigen may also be unique for each protein, as the secondary and tertiary structure depend on intra and intermolecular interactions. Although the BSA in this study was used as a model protein for the antigen, the results and current uncertainties highlights the importance of establishing how the effect on protein structure will likely affect the establishment of a protective immune response for each unique antigen. Linking the determinants of antigenicity and protein sequences through machine learning is one such approach which shows promise as an avenue for greater progression in this area [35].

The A549 and DCs indicated a good viability profile towards the hybrid cationic CHL NPs and cationic CHL NPs/NCMPs up to 2.5 mg/mL concentration (Figure 6). The difference at the same concentration between the formulations was due to dilution of the NPs with leucine used in spray drying. However, the probability of a high local concentration being achieved within specific regions of the lungs is unlikely due to dispersion of the inhaled formulation over a wide surface area in the lungs, compared to the concentrated surface area in a 96 well plate [4]. Furthermore, lower doses of hybrid cationic CHL NPs can be used due to their enhanced interaction with negatively charged proteoglycans on the surface of cell membranes in comparison with anionic or neutral NPs [17]. This was shown in a study by Kwon et al, comparing cationic and neutral CHL NPs loaded with ovalbumin as model antigen, and demonstrating greater uptake by bone marrow DCs with cationic NPs, but comparable cell viability at 125–250 µg/mg concentration [38].

The potential for the hybrid cationic CHL NPs to induce immunostimulatory responses is an important aspect for its use in vaccine or immunotherapeutic applications. The interaction of the NPs with JAWS II cells, which is a murine DC line, can offer insight into the degree of uptake and immunogenicity. The internalization of hybrid cationic CHL NPs by the cells after 1 h of co-incubation was demonstrated visually by CLSM (Figure 7A,B), and was further supported by the flow cytometry data, which showed the particulate FITC-BSA associated with the cells at a significantly higher quantity compared to FITC-BSA in solution (Figure 8). The result is not surprising, as other studies have demonstrated similar phenomena regarding the uptake of polymeric nanoparticles by DCs, and confirms the first step relevant for initiating an adaptive immune response [39]. The observed temperature dependent uptake could be associated with energy dependent endocytosis and the increased association of the particles with the cells seen through microscopy and flow cytometry (Figure 7 and Figure 8) does indeed involve cell uptake as opposed to simple adsorption to the cell surface [16].

The particle internalization by DCs is in line with the subsequent activation of the cells seen through upregulation in the CD40, CD86, and MHC-II cell surface markers. The CD40 and CD86 are generally considered to be indicative of DC activation and are involved in further co-stimulatory T cell interactions [40]. It was interesting to note the difference in the degree of cell marker expression compared to the LPS, which is a group of molecules located in the membrane of gram-negative bacteria, and is known to bind to the toll like receptors to initiate immune responses [41]. The LPS induced a significantly higher increase in CD86 compared to the NPs, whereas the CD40 were comparable between the groups (Figure 9). The difference could be due to differences in the characteristics of DC activation or differences in the kinetics of surface marker upregulation. It is unclear how much influence the degrees of CD40 and CD86 upregulations has on the end immune response. However the interaction of both CD40 with CD40L and CD86 with CD28, on the DCs and T cells, respectively, are also thought to be an important co-stimulatory step in the activation of T cells [42,43]. MHC-II is also known to be upregulated in activated DCs through increases in MHC-II synthesis and trafficking, and follows a similar pattern to CD86 [44].The MHC-II molecules are important for the presentation of foreign peptide antigens to T cells which are specific for that particular antigen [44].The antigen specific activation of T cells is an essential aspect of adaptive immune responses and it would be reasonable to assume the relevance of these results, in vitro, in vaccine applications. Establishing a clearer correlation between the in vitro expression of these surface markers and the degree of immune response in vivo would be a helpful for the investigation of potential vaccine formulations.

The data, as a whole, support the possibility of using cationic CHL NPs/NCMPs as carriers for pulmonary vaccine delivery, based on aspects of inhalation, antigen incorporation, uptake of NPs by DCs, and subsequent upregulation of cell surface activation markers. However, in order to further confirm this potential, the platform must be tested in vivo to establish the induction of protective effects. Studies investigating late-stage biological outcomes such as serum antibody production and memory cell responses could further support the practical use of this formulation.

## 4. Materials and Methods

### 4.1. Materials

The polymer poly(glycerol adipate-co-ω-pentadecalactone) (PGA-co-PDL, MW 16.7 KDa) was synthesized as described previously [25]. Chitosan hydrochloride (CHL) (MW 200,000–400,000); degree of deacetylation (80–95%) was purchased from Heppe Medical Chitosan GmbH, Germany. Fluorescamine was purchased from Acros Organics; Morris Plains, NJ, USA. Phosphate buffered saline tablets pH 7.4 was obtained from Oxoid, UK. Poly (vinyl alcohol) (PVA, MW 13–23 kDa, 87–89% hydrolyzed), bovine serum albumin (BSA), FITC-BSA, RPMI-1640 medium with sodium bicarbonate and L-glutamine, 3-4, 5-dimethylthiazol-2-yl-2,5-diphenyl tetrazolium bromide (MTT), lipopolysaccharide (LPS), and L-leucine were obtained from Sigma–Aldrich, UK. Dichloromethane (DCM), dimethyl sulfoxide (DMSO), tissue culture flasks (75 cm^2^) with vented cap, antibiotic-antimycotic (100×), micro BCA Protein Assay Kit, chambered coverglass (8 well), TrypLE, and 96-well flat bottom plates were purchased from Fisher Scientific, UK. Heat inactivated fetal calf serum (FCS) was obtained from Biosera UK. Immature DCs; (JAWS II), adenocarcinomic human alveolar basal epithelial cells (A549) were purchased from LGC standards, UK.

### 4.2. Methods

#### 4.2.1. Preparation of Hybrid Cationic Chitosan Hydrochloride Nanoparticles

A double emulsion solvent evaporation method was used. Briefly, the internal aqueous phase (0.5 mL) consisted of the BSA solution with surfactant (PVA, 1% *w/v*), and was emulsified in organic phase, DCM, containing PGA-co-PDL (50 mg/mL), in an ice bath, using a probe sonicator (VC X 500 Vibra-CellTM; Sonics & Materials, Inc., Newtown, Connecticut; 13 mm probe). To form double emulsion, the prepared single emulsion was emulsified into external aqueous phase (EAP, 25 mL) containing surfactant (1% *w/v* PVA) using the same probe sonicator followed by 2 h of magnetic stirring to evaporate the organic solvent. To prepare the hybrid cationic CHL NPs, CHL (2, 4, 6, 8, 10, 16, 20 mg/mL) was added to the EAP, to render the NPs positive, at room temperature with stirring for 2 h. Control cationic NPs were produced as above without BSA. For investigation of NPs uptake by DCs and A549 cells, FITC-BSA was added to the internal aqueous phase to facilitate visualization under confocal microscopy (CLSM). The NPs formed were purified by centrifugation (Sigma 3–30k; SIGMA Laborzentrifugen GmbH, Osterode am Harz, Germany) at 30,000× *g* for 1 h at 4 °C, and the sediment was resuspended in distilled water and washed twice.

#### 4.2.2. Quantification of Chitosan Hydrochloride Adsorption

The interaction between fluorescamine and the amino groups of CHL leads to fluorescence and was used to quantify CHL adsorbed onto NPs [45]. One hundred microliters of Fluorescamine/DMSO solution (0.2%) was added to a black 96-well fluorescent microplate containing CHL supernatant collected during wash (20 µL). The plate was incubated for 3 h in dark followed by fluorescence measurements using a microplate reader (CLARIOstar^®^ BMG Labtech, Germany) at 390/515 nm (excitation/emission wavelength). The extent of CHL adsorbed onto the NPs was determined from Equation (1):

(1) $$q=\frac{(C_{i}-C_{e\;})\;}{W}V$$

where *q* is the mass of CHL (mg) adsorbed on to the NPs, *C_i_* is the initial and *C_e_* the free CHL concentration (mg/mL), *W* is the mass of NPs (mg), and *V* is the suspension volume (mL). A standard curve was produced. The adsorbed CHL was determined following subtraction of un-adsorbed (free) CHL in supernatant from the initial CHL used in the preparation.

#### 4.2.3. Adsorption Isotherms Models

The adsorption mechanism of CHL onto NPs was determined using the following isotherm models: Langmuir [18], (Equation (2)); BET (Brunauer–Emmett–Teller) [46], (Equation (3)); Freundlich [19], (Equation (4)); and Halsey [47], (Equation (5)). The quantified experiment adsorption data was applied to the isotherm models, and a linear plot was obtained. Here, *q* is the mass of CHL adsorbed onto NPs, *C_e_* is the free CHL concentration, *q_m_* and *k* are the adsorption capacity constants, and *b* and *n* are the intensity of adsorption constants:

(2) $$\frac{C_{e}}{q}=\;\frac{1}{b\;q_{m}}+\frac{C_{e}}{q_{m}}$$

(3) $$\frac{C_{e}}{q-\left(1-C_{e}\right)}=\;\frac{1}{b\;q_{m}}+\frac{b-1}{b\;q_{m}}$$

(4) $$\log q=\log k+\;\frac{1}{n\;}\log C_{e}$$

(5) $$\ln q=\frac{1}{n}\ln k-\;\frac{1}{n\;}ln\;(-\ln C_{e})$$

#### 4.2.4. Characterization of Nanoparticles

- Size, Zeta Potential, and Polydispersity Index (PDI)

The particle size, zeta potential, and PDI were ascertained by dynamic light scattering using a Zetasizer Nano ZS (Malvern Instruments Ltd, UK). The sample was placed into a cuvette diluted with deionized water and the readings were performed at 20 °C, with the attenuator and measurement settings on automatic (average of three measurements of three independent batches ± SD).

- Encapsulation efficiency and protein loading

The BSA encapsulated into NPs was determined by analyzing the supernatant (obtained after two washes by centrifugation at 82,000× *g* for 25 min) using the microBCA assay (*n*=3, ± SD) at 562 nm according to manufactures directions. The encapsulation efficiency (EE %) and drug loading (DL) were determined using Equations (6) and (7) below:

(6) $$EE\;\%\;=\;\frac{amount\;of\;BSA\;added-free\;amount\;of\;BSA}{amount\;of\;BSA\;added}\times 100$$

(7) $$DL\;=\;\frac{actual\;amount\;of\;encapsulated\;BSA\;\left(µg\right)}{actual\;amount\;of\;nanoparticles\;\;\left(mg\right)}$$

#### 4.2.5. Nanocomposite Microparticles Production by Spray Drying

The mixture of hybrid cationic CHL NPs and L-leucine solutions (ratio 1:1.5 *w/w*) were spray dried to produce nanocomposite microparticles (NCMPs) using a Büchi B-290 mini spray dryer with a standard 0.7 mm diameter two-fluid nozzle and high efficiency cyclone (Büchi Labortechnik, Flawil, Switzerland). The spray drying conditions consisted of: feed rate 10%, aspirator capacity 100%, atomizing air flow 400 L/h, inlet temperature 100 °C, with outlet temperature between 42–46 °C, and feed concentration 12.5 mg/mL. The NCMPs powder was sampled from the high efficiency cyclone and placed in a desiccator at room temperature until further use.

#### 4.2.6. Characterization of Nanocomposite Microparticles

- Morphology and particle size

The morphology of the cationic CHL NPs/NCMPs was qualitatively investigated by placing the sample on top of carbon aluminum stubs, coated with palladium for 3 min at 25 mA (EmiTech K 550X Gold Sputter Coater) and examined using scanning electron microscopy (FEI Quanta^TM^ 200, the Netherlands). For measurement of the size of cationic CHL NPs from the NCMPs, cationic CHL NPs/NCMPs (5 mg) were resuspended in deionized water (10 mL), placed into a cuvette and measured at room temperature as described above (4.2.4).

- Theoretical aerodynamic diameter

The tap density of cationic CHL NPs/NCMPs (0.2 g) was determined by adding the powder sample to a 5 mL measuring cylinder, tapped 100 times until no reduction in volume was noted (*n* = 3). The theoretical aerodynamic diameter (*d_ae_*) was determined from Eq. 8, in which *d* is geometric particle size and *ρ* is tap density:

(8) $$\begin{array}{c} d_{ae\;}=d\;\sqrt{\frac{\rho }{\rho_{1}}}\\ \rho_{1}=1\;g/{\mathrm{cm}}^{3} \end{array}$$

#### 4.2.7. In Vitro Aerosolization Analysis

Cationic CHL NPs/NCMPs were weighed (15 mg dry powder per capsule) and filled into six hydroxypropyl methylcellulose capsules (size 3). The capsules were placed into an 8-pin Cyclohaler^®^ and aerosolized (60 L/min for 4 s) into the Next Generation Impactor (NGI) with the plates of each stage coated with a solution of tween 80: acetone (1% *w/v*). Following aerosolization, the samples were collected with a known volume of DCM/0.15 M NaCl mixture (2:1) to separate the PGA-co-PDL from BSA which was determined as described above (encapsulation efficiency and protein loading section) (*n* = 3). The fine particle dose (FPD, µg) and fine particle fraction (FPF%) were calculated as the mass and percentage of emitted dose collected from stages with *d_ae_* ≤ 4.46 μm, respectively. Log-probability analysis was used to calculate the mass median aerodynamic diameter (MMAD, μm).

#### 4.2.8. In Vitro Release Study

Cationic CHL NPs/NCMPs samples (15 mg) were dispersed in microtubes containing 1.2 mL PBS buffer (pH 7.4) rotating in a HulaMixer at 20 RPM (Fisher Scientific, UK), incubated at 37 °C. At given timepoints (0, 2, 4, 6, 20, 24, 48 h), the microtubes were centrifuged (accuSpin Micro 17, Fisher Scientific, UK) at 17,000× *g* for 30 min and 0.5 mL of supernatant was collected for analysis, and the sample deposits were resuspended in fresh buffer. Analysis of the sample was performed using the microBCA protein assay (*n* = 3), and the cumulative protein released determined from Equation (9):

(9) $$\%\;Cumulative\;protein\;released=\frac{cumulative\;protein\;released}{protein\;loaded}\times 100$$

The release mechanism was determined from graphical plots of the following kinetic models: zero order, first order, and Higuchi with the correlation coefficient closest to ‘1′ used as the mechanism and order.

#### 4.2.9. Analysis of BSA Structure

SDS-PAGE: The fragmentation of BSA was examined by SDS-PAGE gel electrophoresis. Samples (10–20 mg) were suspended in PBS buffer (pH 7.4, in 0.5–1.5 mL) and the BSA was released according to in vitro release study method above. Fifty µL of supernatant was taken and added to SDS-PAGE (CVS10D omniPPAGE vertical gel electrophoresis system. Geneflow Limited, UK) and 75 µg/mL of BSA in water was used as a control. The samples and standard were mixed with protein loading buffer (1:1) for 3 min at 95 °C and loaded into wells (25 µL per well). The SDS-PAGE was operated at 100 V for 2.3 h, and stained with Coomassie Brilliant Blue followed by de-staining in water, and imaged using a Molecular Imager^®^ Gel Doc^™^ XR+ System.

Circular dichroism (CD): The secondary structure of BSA released (48 h) and standard BSA (as a control) was evaluated at 20 °C using CD spectropolarimeter (J-815, Jasco, UK) [48]. Five scans per sample (speed 50 nm/min) were completed applying a path-length cell (10 mm), far-UV wavelength (260–180 nm), data pitch (0.5 nm), and band width (1 nm). A baseline measurement was performed and subtracted from data obtained from the samples [49]. All parameters for analysis between BSA release and standard were the same. The CDSSTR method [50] and protein reference set 3 from the DichroWeb server [51] were used to evaluate the percentage content of the secondary structure of the BSA. The Dichroweb site was used to analyze the percentage composition of α- helical, β- strands, turns, and unordered secondary structures.

#### 4.2.10. Cell Viability Studies

An MTT assay was applied to determine the cell viability of A549 (cultured in complete medium: RPMI-1640, 10% FCS, 1% antibiotic/antimycotic solution) and immature DCs (cultured in complete medium: MEM alpha incorporating 4 mM L-glutamine, 1 mM sodium pyruvate, 20% FCS, 1% antibiotic/antimycotic solution, and 5 ng/mL recombinant murine GM-CSF) after incubation with hybrid cationic CHL NPs and cationic CHL NPs/NCMPs over 24 h. Cells (2.5 × 10^5^ cells/mL) were seeded in 96-well plates with culture medium (100 µL) incubated at 37 °C, 5% CO_2_, for 24 h. The wells were replaced with samples (0–5 mg/mL) in fresh medium (100 µL) (*n* = 3) and incubated for a further 24 h as above. MTT solution (40 µL, 5 mg/mL) was placed in each well and incubated as above for 2 h. This was followed by removal of media and the formazan crystals were dissolved in DMSO (100 μL). The absorbance (570 nm) of solubilized dye was determined using an Epoch microplate reader (BioTek, UK). The cell viability (%) was evaluated as the absorbance ratio of treated-NPs or NCMPs to non-treated cells (control).

#### 4.2.11. Nanoparticle Uptake by DCs Using Confocal Microscopy

Hybrid cationic CHL NPs incorporating FITC-BSA were prepared for visualization of DCs uptake. DCs were seeded at a density of 2 × 10^5^ cells/400 µL in an 8-well chamber cover-glass and incubated at 37 °C, 5% CO_2_, for 48 h. This was followed by addition of FITC-BSA loaded cationic CHL NPs (40 µg/40 µL) and incubated for 1 h under the same conditions. The media was carefully removed, and the cells were fixed using 300 μL of 4% paraformaldehyde/PBS for 15 min then washed with PBS. Cell membrane and nuclei staining: 100 µL WGA TR (5 µg/mL) (red, cell membrane) and 100 µL of DAPI (blue, nuclei) were inserted into the wells and incubated as above for 10 min. PBS was then used to wash the cells. The uptake by DCs was imaged using confocal microscopy (Carl Zeiss LSM 710, UK) fitted with a Plan Neofluar 63×/0.30 numerical aperture (NA) objective lens at excitation wavelength of 595 nm (red channel: WGA TR), 358 nm (blue channel: DAPI), and 488 nm (green channel: FITC-BSA). Zeiss LSM software was used for image analysis.

#### 4.2.12. Nanoparticle Uptake/Adherence by DCs Using Flow Cytometry

Nanoparticle uptake/adherence by the DCs was also evaluated using flow cytometry. DCs were prepared in the same manner as 4.2.11, followed by the addition of FITC-BSA-loaded hybrid cationic CHL NPs (40 µg/40 µL) or FITC-BSA at the same loaded concentration as the cationic CHL NPs (determined in 4.2.4). Media FITC-BSA and FITC-BSA-loaded hybrid cationic CHL NPs were incubated for 1 or 4 h at 37 °C. The un-adhered cells were collected first, followed by the adhered cells, which were treated with TrypLE for 5 min at room temperature. The collected cells were pooled, centrifuged at 200× *g* for 5 min followed by two washes with PBS. This was followed by resuspension of cells which were incubated in PBS containing 7AAD (abcam, Cambridge, UK) for 10 min, then analyzed using a BD Accuri C6 flow cytometer (BD, Wokingham, UK).

#### 4.2.13. DC Activation by Nanoparticles

The upregulation of surface markers on the DCs were measured to determine the immunostimulatory effects of the nanoparticles. DCs were prepared and BSA loaded hybrid cationic CHL NPs particles were added in the same manner as 4.2.12, and incubated for 24 h. Control groups consisted of blank media (negative) and LPS (100 ng/mL) (positive). The cells were collected and washed as in 4.2.12 and stained with anti-mouse FITC-CD40, PE-CD86, and APC-MHC-II (BD, UK). The cells were washed and stained with 7AAD for 10 min before being measured using a BD Accuri C6 flow cytometer.

#### 4.2.14. Statistical Analysis

The results are presented as mean ± SD. Statistical analysis was performed using one-way analysis of variance (ANOVA) with Tukey’s comparison, with significance indicated at *p* < 0.05 or otherwise specified.

## 5. Conclusions

Adsorption of CHL onto the surface of PGA-co-PDL NPs can be exploited as an effective strategy to create positively charged NPs, which have potential for immunostimulation in vaccine applications. The preparation method used successfully produced hybrid cationic CHL NPs, and the multilayer surface adsorption of CHL conformed to the Freundlich model indicating cooperative adsorption. The incubation of hybrid cationic CHL NPs with dendritic cells (DCs) resulted in particle uptake within 1 h of incubation, as shown by confocal microscopy and flow cytometry. Upregulation of CD40, CD86, and MHC-II cell surface markers after incubation also indicated that DCs were being activated by the particles. The formulation exhibited a low toxicity profile and could be prepared into a dry powder form for inhalation. The data obtained in this investigation indicate that cationic CHL NPs/NCMPs exhibit potential for the application of delivering encapsulated protein to the dendritic cells of the lung and inducing immunostimulatory responses for adaptive immunity. Future studies will investigate this delivery system with vaccine candidates and tested in vivo in appropriate rodent models.

## Figures and Tables

**Table 1:** Adsorption parameters obtained by linear regression from the isotherm models.

Isotherm Equations	Adsorption Capacity	Adsorption Intensity	Regression Coefficient
Langmuir *	*q_m_* = 1.2366	*b* = −0.2149	0.9167
Langmuir	*q_m_* = 2.28	*b* = 0.146	0.8415
BET	*q_m_* = 0.4288	*b* = 0.8561	0.0015
Freundlich	*k* = 0.2893	*n* = 0.5434	0.9988
Halsey	*k* = 1.9422	*n* = 0.5406	0.9974

* CHL/PGA-co-PDL NPs made at 0–8 mg/mL CHL initial concentration. Langmuir */Langmuir *q_m_* units—mg/g and BET *q_m_*—mol/g.

**Table 2:** In vitro kinetic model parameters of BSA released from cationic CHL NPs/NCMPs.

Formulation	Zero Order		First Order		Higuchi	
	r^2^	k_o_ (h^−1^)	r^2^	K_1_ (h^−1^)	r^2^	k_1_ (h^−½^)
Cationic CHL NPs/NCMPs	0.777	1.218	0.919	−0.016	0.928	9.316

**Table 3:** The BSA secondary structure released from cationic CHL NPs/NCMPs compared to standard (*n* = 3).

BSA	Helix	Strands	Turns	Unordered
Standard	51.5 ± 0.007	21.5 ± 0.007	9.0 ± 0	17.5 ± 0.007
Released	43.0 ± 0.007	29.0 ± 0.014	7.0 ± 0	20.5 ± 0.007

## Data Availability

The data presented in this study are available in this article.
